# Editorial: Linguistic Influences on Mathematics

**DOI:** 10.3389/fpsyg.2016.01035

**Published:** 2016-07-12

**Authors:** Ann Dowker, Hans-Christoph Nuerk

**Affiliations:** ^1^Experimental Psychology, University of OxfordOxford, UK; ^2^Department of Psychology, University of TuebingenTuebingen, Germany; ^3^Knowledge Media Research Center, University of TuebingenTuebingen, Germany; ^4^LEAD Graduate School and Research NetworkTuebingen, Germany

**Keywords:** language, numerical cognition, psychology of arithmetic, verbal counting systems, cross-linguistic research

For many years, an abstract, amodal semantic magnitude representation, largely independent of verbal linguistic representations, has been viewed as the core numerical or mathematical representation (Dehaene and Cohen, 1995). This assumption has been substantially challenged in recent years (e.g., Miura and Okamoto, 2003; Nuerk et al., 2004, 2005; Dowker et al., 2008; Colomé et al., 2010; Helmreich et al., 2011; Krinzinger et al., 2011; Pixner et al., 2011a,b; Göbel et al., 2014; Imbo et al.; Klein et al.). Linguistic properties affect not only verbal representations of numbers (Seron and Fayol, 1994; Zuber et al., 2009; Pixner et al., 2011a), but also numerical magnitude representation (Nuerk et al., 2005; Pixner et al., 2011b), spatial magnitude representations (Shaki et al., 2008; Helmreich et al., 2011), calculation (Colomé et al., 2010; Krinzinger et al., 2011; Göbel et al., 2014), parity representation (Iversen et al., 2004, 2006; Nuerk et al., 2004), place-value representation (Miura and Okamoto, 2003; for a review, see Nuerk et al.) and even early number acquisition (Sarnecka, this issue). Thus, we postulate that numerical and arithmetic processing are not fully independent of linguistic processing. This is not to say, that in patients, magnitude processing cannot function independently of linguistic processing (e.g., Dehaene and Cohen, 1997), we just suppose, these functions are connected in the functioning brain. So far, much research about linguistic influences on numerical cognition has simply demonstrated that language influences number without investigating the level at which a particular language influence operates. Here we want to distinguish several linguistic levels at which numerical processing may be influenced, according to which we group the articles in our special issue:

- *Conceptual*: Conceptual properties of language
- *Syntactic*: The grammatical structure of languages beyond the word level influences
- *Semantic*: The semantic meaning or existence of words
- *Lexical*: The lexical composition of words, in particular number words
- *Visuo-spatial-orthographic*: Orthographic properties, such as the writing/reading direction of a language.
- *Phonological*: Phonological/phonetic properties of languages
- *Other language-related skills*: Verbal working memory and other cognitive skills related to language representations

## Conceptual influences

Beyond single phonemes, graphemes, words and sentences, linguistic structures are also shaped by linguistic concepts. The linguistic markedness concept suggests that for (almost) each adjective pair, a ground (unmarked) form and a derived (marked) form exist (e.g., efficient and inefficient; marked by “in”).

We consider the markedness concept “conceptual” (see Nuerk et al., 2004). However, many language models do not consider a conceptual level as such and often the lexical or semantic level is the highest level. Levelt et al. (1999), however, proposed a conceptual level in the language production model. It is the highest level in this model and is assumed to be involved in the conceptual preparation of lexical concepts. In Nuerk et al. (2004, p.859), we suggested that linguistic markedness could operate at just such a conceptual level and that other verbal influences like phonological ones will operate at a different (lower) level, e.g., the phonological encoding in the mental lexicon.

Numbers possess several attributes, which can be distinguished into unmarked ground form (large, even, divisible) and marked form (small, odd, indivisible; Hines, 1990). As regards spatial organization “right” is unmarked and “left” is marked (Nuerk et al., 2004). Usually responses are faster, when markedness of stimuli and responses are congruent (e.g., left-odd, right-even). Schroeder and Pfister (this issue) investigated SNARC and MARC effects on card distribution to fellow card players. They observed markedness effects in that magnitude and parity influence card distribution. However, in this natural setting, the markedness effect is inverted to a normal parity judgment task, extending earlier findings in deaf signers (Iversen et al., 2004), and left-handers (Huber et al., 2015). This implies that not only bodily, but also task-specific constraints need to be taken into account, when linguistic effects on mathematical cognition on the construct level are examined.

## Syntactic influences

Number processing in real life situations occurs in natural language and is described by grammatical number. (i.e., singular for 1 and plural for numbers 2 and greater in English). Languages differ substantially in their use of grammatical number (see Overmann, 2015) analysis of 905 languages): For instance, 7% of these languages lacked grammatical number altogether despite having lexical numbers. Influences of grammatical numbers on numerical cognition have been shown in two effects. First, Roettger and Domahs (2014) observed a grammatical SNARC effect: singular inflected words elicited faster responses on the left hand side and plural inflected words on the right Second, as beautifully outlined by Sarnecka's (this issue) review, the sheer existence of certain grammatical number enhances development of number concepts in children. In languages without differentiation between singular and plural, the development of number understanding in children is later. Moreover, grammatical distinction between singular, dual (a grammatical form for “two”) and plural present in several languages further enhances, yet partially hinders number development in children. In some cases, the syntactic structure of a language both influences development of numerical understanding and spatial mappings of numbers.

## Semantic influences

Word meanings also influence numerical or arithmetic processing. Daroczy et al. reviewed text problems and found that numerical properties and semantic properties are often interacting. For example, the consistency effect suggests that text problems are easier, when the required operation is consistently associated with the semantics of the words. For instance, addition is more associated with “more,” “buy,” “get,” etc., while subtraction is more associated with “less,” “sell,” “give,” etc. When text problems are presented in a way that makes such associations misleading, children and adults perform less well. This highlights an interrelation between word meaning and preferred arithmetic operations.

## Lexical influences

Most of the papers in our special issue as well as in the literature are concerned with lexical influences, in particular number words. In general, a transparent number word structure seems to help numerical performance even for problems not involving number words (Nuerk et al., 2015). Two types of lexical influences are discussed in our special issue. The first involves the inversion property. Some languages like Arabic, Dutch and German invert the order of tens and units (“one-and-twenty” for 21), which creates problems in several tasks. Moeller et al. (this issue) compared transcoding (writing numbers to dictation) skills in Japanese and German. The Japanese children did much better. In particular, Japanese children make far fewer inversion errors; but also fewer errors in general. Xenidou-Dervou et al. (this issue) show that the inversion property does not affect all numerical and arithmetic skills. Dutch children (with inversion) lag behind English children in symbolic but not non-symbolic arithmetic. A working memory overload in Dutch was found in non-symbolic, but not symbolic magnitude. However, as Bahnmueller et al. (this issue) show, inversion effects do not even affect all aspects of symbolic number processing. While children's and adults' two-digit Arabic number comparison is influenced by inversion properties of a language, adults' three-digit Arabic number comparison is not. Moreover, van Rinsveld et al. (this issue) found that inversion affected complex but not simple symbolic arithmetic in German-French bilingual secondary pupils. Finally, Prior et al. (this issue) gave Hebrew-Arabic bilinguals oral arithmetic problems, because Arabic but not Hebrew number words possess the inversion property. Participants solved arithmetic problems best when the language structure corresponded to the arithmetic problem. This implies that—contrary to earlier claims—L1 does not completely dominate arithmetic processing, but that both L1 and L2 shape numerical and arithmetic.

The second line of research at the lexical level is power transparency. Unlike most European languages, most Asian languages are extremely transparent with respect to the power of a given number (e.g., “ten-two” for 12). From 11 on, children and adults can derive the power of each number directly from the number word. It has been argued that this transparency may be responsible for Asians' better skills at counting, representing 2-digit numbers, and general arithmetic (Miller et al., 1995; Miura and Okamoto, 2003). However, such results are confounded by the many other educational and cultural differences between countries. One way of obtaining more specific evidence of language effects is to compare children studying in different languages in the same country and educational system. For instance, the Welsh counting system, unlike the English system, is transparent. Dowker et al. (2008) found that children in Welsh-medium primary schools did not do better in arithmetic overall, but showed specific advantages in reading and comparing two-digit numbers. Extending those results Dowker and Roberts observed that Welsh-medium children give more precise and consistent representations of 2-digit numbers on empty number line tasks. Mark and Dowker studied children in Chinese and English medium primary schools in Hong Kong. The Chinese medium children were better at some tasks but not others: e.g., they were better at counting backwards but not forwards; and were not better at number comparison. Thus, we can conclude that lexical influences do affect arithmetic, but not as pervasively as sometimes assumed.

## Visuo-spatial-orthographic influences

Visual-spatial-orthographic influences mostly involve the reading/writing direction of a given script or its complexity. Usually, space-number relations are associated with the dominant reading/writing direction (for a review see Fischer and Shaki, 2014). However, reading/writing direction already influences spatial-numerical directionality, before children can read or write (Patro and Haman, 2012; Nuerk et al., 2015). Most studies so far have investigated visuo-spatial-orthographic influences on the horizontal left/right dimension. Göbel (this issue) showed that cultural influences on number-space-relations also include the vertical dimension. Fischer and Shaki (this issue) proposed two steps in the shaping of directional space-number representations in adults: “*the spatial dimension selected for mapping of numbers reflects the stimulus and response features of the current task”* and “*the orientation of the SNA is influenced by spatial experience.”*

Relatedly, Rodic et al. examined whether learning spatially complex scripts (e.g., Chinese) is related to mathematical performance. They found no evidence that exposure to a spatially complex script improves mathematics.

We conclude that visuo-spatial orthographic skills seem to shape the direction of space-number relations, but not arithmetic skills themselves.

## Phonological influences

Jordan et al. examined phonological skills in children with difficulties in reading, mathematics or both and found minor influences on phonology on mathematics. Pixner et al. (this issue) examined children with cochlear implants (CI), who usually have phonological (and also other) language deficits. They found general deficits in such children in multiplication, subtraction and number line estimation, but specific deficits in (verbally mediated) place-value manipulation. We conclude that phonological skills are not related to mathematical functioning *per-se*, but to verbal representations/manipulations of number.

## Other language-related skills: verbal working memory and other cognitive skills

Verbal working memory is associated with complex arithmetic since Ashcraft and Stazyk (1981) seminal paper. Soltanlou et al. (this issue) investigated whether verbal or spatial working memory influences multiplication skill most strongly. They observed an age-related shift from verbal WM to spatial WM influences over time. Thus, working memory data from adults or one children age-group are not representative for its influence in different developmental stages.

## Summary

Linguistic influences on number processing are ubiquitous. They occur at conceptual, semantic, syntactic, lexical, visuo-spatial-orthographic, phonological, and other levels. Research should now address more precisely which language characteristics at which level influence particular numerical tasks at particular ages.

## Author contributions

All authors listed, have made substantial, direct and intellectual contribution to the work, and approved it for publication.

### Conflict of interest statement

The authors declare that the research was conducted in the absence of any commercial or financial relationships that could be construed as a potential conflict of interest.
